# Beclin 1 Deficiency Correlated with Lymph Node Metastasis, Predicts a Distinct Outcome in Intrahepatic and Extrahepatic Cholangiocarcinoma

**DOI:** 10.1371/journal.pone.0080317

**Published:** 2013-11-26

**Authors:** Tian-Tian Wang, Qing-Hua Cao, Ming-Yuan Chen, Qing Xia, Xin-Juan Fan, Xiao-Kun Ma, Qu Lin, Chang-Chang Jia, Min Dong, Dan-Yun Ruan, Ze-Xiao Lin, Jing-Yun Wen, Li Wei, Xing Li, Zhan-Hong Chen, Lei Wang, Xiang-Yuan Wu, Xiang-Bo Wan

**Affiliations:** 1 Department of Medical Oncology, the Third Affiliated Hospital, Sun Yat-sen University, Guangzhou, China; 2 Gastrointestinal Institute, the Sixth Affiliated Hospital of Sun Yat-sen University, Guangzhou, China; 3 Department of Pathology, the First Affiliated Hospital, Sun Yat-sen University, Guangzhou, China; 4 State Key Laboratory of Oncology in South China, Sun Yat-sen University Cancer Center, Guangzhou, China; 5 Department of Oncology, Shanghai Jiao Tong University affiliated First People’s Hospital, Shanghai, China; 6 Guangdong Provincial Key Laboratory of Liver Disease Research, Sun Yat-sen University, Guangzhou, China; University of North Carolina School of Medicine, United States of America

## Abstract

Autophagy can be tumor suppressive as well as promotive in regulation of tumorigenesis and disease progression. Accordingly, the prognostic significance of autophagy key regulator Beclin 1 was varied among different tumors. Here, we detected the clinicopathological and prognostic effect of Beclin 1 in the subtypes of intrahepatic cholangiocarcinoma (ICC) and extrahepatic cholangiocarcinoma (ECC). Beclin 1 expression level was detected by immunohistochemistry staining in 106 ICC and 74 ECC patients. We found that Beclin 1 was lowly expressed in 126 (70%) cholangiocarcinoma patients, consist of 72 ICC and 54 ECC. Moreover, the cholangiocarcinoma patients with lymph node metastasis (N1) had a lower Beclin 1 level than that of N0 subgroup (*P=0.012*). However, we did not detect any correlations between Beclin 1 and other clinicopathological features, including tumor subtypes, vascular invasion, HBV infection, liver cirrhosis, cholecystolithiasis and TNM stage. Survival analysis showed that, compared with the high expression subset, Beclin 1 low expression was correlated with a poorer 3-year progression-free survival (PFS, 69.1% VS 46.8%, *P*=041) for cholangiocarcinoma. Importantly, our stratified univariate and multivariate analysis confirmed that Beclin 1 lowly expressed ICC had an inferior PFS as well as overall survival than ECC, particularly than that of Beclin 1 highly expressed ECC patients. Thus, our study demonstrated that Beclin 1low expression, correlated with lymph node metastasis, and might be a negative prognostic biomarker for cholangiocarcinoma. Combined Beclin 1 level with the anatomical location might lead to refined prognosis for the subtypes of ICC and ECC.

## Introduction

Cholangiocarcinoma is a malignant neoplasm in the biliary duct system accounts for 10-25% of primary hepatic tumor and represents 3% of gastrointestinal cancer worldwide [1,2]. Anatomically, cholangiocarcinoma can be dichotomized into intrahepatic cholangiocarcinoma (ICC) or extrahepatic cholangiocarcinoma (ECC, including hilar cholangiocarcinoma) according to their location [3]. Although radical surgery plus adjuvant chemotherapy produce favorable prognosis for early stage subgroup, regional invasiveness and distant metastasis remains the major cause of high cancer mortality for advanced cholangiocarcinoma patients [4]. Molecular alterations in oncogenes, tumor suppressor genes, cell-cycle regulators and growth factors, are attributing to the development and progression of cholangiocarcinoma. Supported by prognostic biomarkers, such as PCNA [5], CD133 [6], Skp2 [7], LAPTM4B-35 and Her2/neu [8,9], the cholangiocarcinoma prognosis was defined more accurately. Overexpression of EGFR, for example, was occurred in 27.4% ICC subgroup and 19.2% ECC subgroup, predicted a poor outcome as well as a high risk to tumor recurrence [10]. EGFR monoclonal antibody Cetuximab plus GEMOX chemotherapy displayed a 63.0% objective response for advanced cholangiocarcinoma patients [11]. Thus, identifying more EGFR-alike molecular markers, that not only predict the prognosis more accurately but also direct therapeutic regimen selection, will be of great survival benefit for cholangiocarcinoma patients. 

Autophagy is a cellular degradation process that captures and digests intracellular proteins or organelles in lysosomes. Autophagy can act as a double-edged sword function of tumor suppression and tumor promotion in cancer initiation as well as progression [12,13]. However, the role of autophagy in the growth, development and relapse of tumor is still poorly understood [14]. As the first identified mammalian autophagy effector, Beclin 1 [15], also known as Atg6, played an essential role both in tumor formation and progression. Allelic loss of *Beclin 1* gene rendered partially autophagy defection and induced spontaneous hepatocellular carcinoma in mice [16]. In human breast, prostate and ovarian tumors, the monoallelical deletion of *Beclin 1* gene was occurred in 40-75% of patients [17-19]. Moreover, blockade of Beclin 1 by siRNA, even under p53 mutation context, could significantly decrease the accumulation of autophagosomes and sensitize resistant breast, pharyngeal, cervical, lung and rectum cancer cells to radiotherapy [20]. Besides to its autophagic role in determination of cancer cell destiny [21]. Beclin 1 was also reported to be a prognostic biomarker in a variety of tumors [22-25]. In hepatocellular carcinoma, Beclin 1 inactivation related autophagy defection was correlated with malignant clinicopathological features, and positive Beclin 1 expression predicted a better overall survival and disease-free survival in a Bcl-X(L)-positive expression backgroun[22].. Moreover, Beclin 1 downregulation was associated with lymph node metastasis and poor outcome in intrahepatic cholangiocellular carcinoma [26].

In the present study, we further detected Beclin 1 expression, and characterized its clinicopathological function in the subtypes of ICC and ECC. We found that Beclin 1 was lowly expressed in cholangiocarcinoma, and correlated with lymph node metastasis. Importantly, Beclin 1 low expression predicted an inferior PFS, and was a negative prognostic biomarker for cholangiocarcinoma. Combining Beclin 1 level with tumor location led to a more accurate prognosis definition for ICC and ECC.

## Patients and Materials

### Patients and eligibility

A total of 194 non-metastatic and histologically confirmed cholangiocarcinoma patients in the Third Affiliated Hospital, the First Affiliated Hospital and Cancer Center of Sun Yat-sen University (Guangzhou, China) from June 2000 to August 2010 were included in the present study. During the microarray construction and immunohistochemistry staining process, 14 cases were excluded for insufficient or detached tissues and 180 cases were brought into this study. All patients received surgery, and part of cases was given adjuvant chemotherapy. Patients were included with the following inclusion criteria: pathologically confirmed as ICC, ECC (including of hilar cholangiocarcinoma); without previously ontological surgery, chemotherapy, or radiotherapy; and all patients had the completed follow-up information and paraffin-embedded specimens. Moreover, patients were excluded for any of the following reasons: previously received any anticancer therapy, prior malignancy, pregnancy, the gallbladder cancer. The patient TNM stage was defined according to 2010 AJCC staging system for cholangiocarcinoma. This study was approved by the Human Ethics Committee of the Third Affiliated Hospital, Sun Yat-sen University. A written informed consent was obtained from all the patients at the time of admission, with which the blood, tissue and other sample were authorized to scientific purpose. 

### Tissue microarray (TMA) construction

Prior to TMA construction, we firstly reviewed the hematoxylin and eosin-stained slides, and chose the tumor zone in the paraffin-embedded specimens. TMA was designed in accordance with the protocol that we previously described [27]. Briefly, two cores from the chosen tumor zone, and additional one core from normal adjacent tissue were used to construct the TMA. Firstly, a hollow needle was utilized to punch the cylinders tissue cores (1.0 mm in diameter) from selected donor tissues. Secondly, the punched tissue was inserted into a recipient paraffin core in a precisely spaced, array pattern, using an automatic TMA instrument (Beecher Instruments, Silver Spring, Maryland, USA) [28]. 

### Immunohistochemical (IHC) staining and semi-quantitative assessment

IHC staining was performed as we previously described [29]. Briefly, the TMA blocks were cut into 4-μm sections, deparaffinised three times in xylene for 30 min and rehydrated with graded alcohols (100% ethyl alcohol for 5 min, 95% ethyl alcohol for 3 min and 75% ethyl alcohol for 3 min). Sections were then heated in antigen retrieval solution (sodium citrate, pH 6.0) in microwave for 15 min, incubated in H_2_O_2_ for 10 min. Thereafter, the TMA sections were incubated at 4°C overnight with primary rabbit anti-Beclin 1 antibody (Santa Cruz, SC-11427) that was diluted at 1:200. In the meantime, negative controls were also utilized by replacing the primary antibodies with non-immune serum immunoglobulin at 1:200 dilutions. The brown granules in cytoplasm or nuclei were regarded as positive staining.

Beclin 1 expression level was evaluated by integrating the percentage of positive tumor cells and the intensity of positive staining. The intensity of staining was scored as follows: negative (score 0), weak (score 1), moderate (score 2), and strong (score 3). We scored the staining extent according to the percentage of positive stained cells in the field: negative (score 0), 0-25% (score 1), 26-50% (score 2), 51-75% (score 3), and 76-100% (score 4). The multiply of the intensity and extent score was considered as the overall IHC score. The IHC score of 6.0, the median IHC score of 0-12.0, was selected as the cutoff point to distinguish Beclin 1 was high or low expression. Immunohistochemical staining level was assessed and scored by two independent pathologists, who were blind to the clinicopathological and follow-up information. 

### Clinical outcome assessment

All patients were followed-up until the date of death or when censored at the latest date (October 30 2012). Overall survival (OS) was defined as the time from diagnosis to the date of death or when censored at the latest date if patients still alive. Progression-free survival (PFS) was defined as the time from diagnosis to the date of local failure/distant metastasis or the date of death or when censored at the latest date.

### Statistical analysis

The correlations between Beclin 1 expression levels and clinicopathological features, including age, gender, subtypes, tumor size, histological type, vascular invasion, HBV infection, liver cirrhosis, cholecystolithiasis, tumor surgery, CEA level, CA125 level, AFP level, CA199 level, tumor stage, node stage and TNM stage, were evaluated by chi-suqare test. The relationships between the Beclin 1 expression level and OS as well as PFS were determined by Kaplan-Meier analysis. The log-rank tests were performed to assess the difference of survival probabilities between patient subgroups. The univariate and multivariate analyses were performed by binary logistic regression model to estimate the hazard ratio and 95% confidence interval. All *P* values quoted were two-sided and *P* < 0.05 was considered as statistically significant. Statistical analysis was performed using SPSS v. 17.0 (SPSS, Inc., Chicago, IL, USA).

## Results

### Patient characteristics and Beclin 1 expression

**Figure 1:**
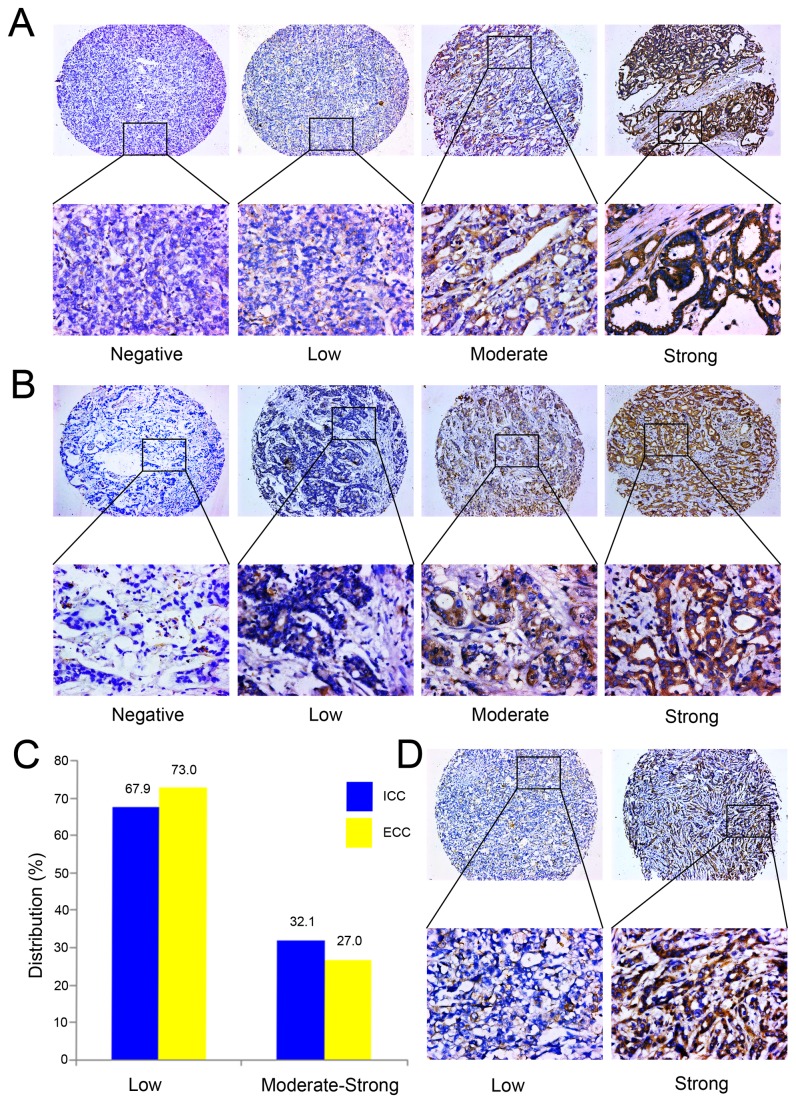
Beclin 1 expression in subtypes of ICC and ECC. Immunohistochemistry analysis of Beclin 1 expression in ICC (A) and ECC (B). Beclin 1 was lowly or moderately expressed in cancer cell cytoplasma, whereas highly expressed in the well differentiated tissue (original magnification, x100). The lower panel displayed the enlarged view (original magnification, x400). (C) The subset patient distribution according to the Beclin 1 expression level in ICC and ECC. (D) Beclin 1 was downregulated in the patients with lymph node metastasis, whereas was upregulated in the subset without lymph node metastasis (original magnification, x100). The lower panel displayed the enlarged view (original magnification, x400).

A total of 180 patients, including of 106 ICC and 74 ECC, that received cholangiocarcinoma resection surgery were recruited in this study. The 1-year, 3-year and 5-year OS ratios were 62.4%, 36.0% and 13.9%, respectively. For PFS, the 1-year, 3-year and 5-year ratios were respectively 71.8%, 61.3% and 39.6%. Immunohistochemistry staining showed that Beclin 1 was lowly expressed in 67.9% (72/106) ICC (Figure 1A) and 73.0% (54/74) ECC (Figure 1B) specimen (Figure 1C), especially in the tumor nest zone, whereas was moderately or strongly expressed in the normal adjacent tissues. 

**Figure 2:**
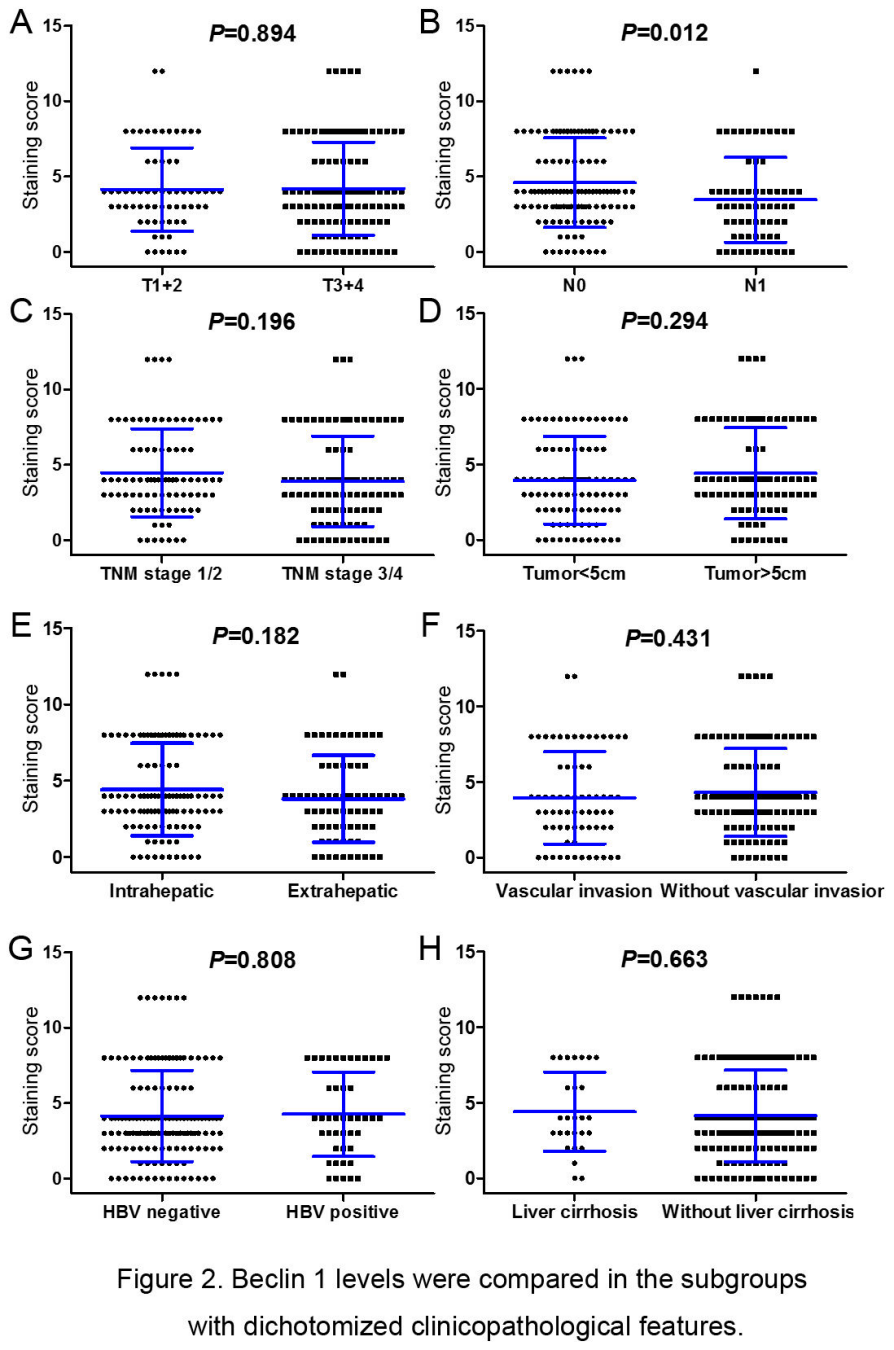
Beclin 1 levels were compared in the subgroups with dichotomized clinicopathological features. For Beclin 1, the features were dichotomized: (A) T stage (T1-2 VS T3-4); (B) N stage (N0 VS N1); (C) TNM stage (1+2 VS 3+4); (D) tumor size (≥5 cm VS <5 cm); (E) tumor subtypes (ICC VS ECC); (F) vascular invasion (with VS without); (G) HBV infection (negative VS positive); (H) Liver cirrhosis (with VS without).

We further detected the relationship between Beclin 1 expression level and clinicopathological features. As summarized in Table 1, when dichotomized the Beclin 1 expression level into high or low, there was no significant correlation between Beclin 1 level and the clinicopathological features (all *P* value > 0.05), such as age, gender, tumor subtypes, tumor size, vascular invasion, HBV infection, liver cirrhosis, cholecystolithiasis, TNM stage. Furthermore, the Beclin 1 immunohistochemistry (IHC) scores were compared in the subgroup with dichotomized clinicopathological features (Figure 2). We found that Beclin 1 IHC score in N0 subgroup (4.60±2.97) was higher than N1 subset (3.45±2.82, *P* =0.012, Figure 1D and 2B). However, we failed to detect a close association between Beclin 1 IHC score and other clinicopathological features, including T stage (T1+2 VS T3+4: 4.14±2.76 VS 4.2±3.08, *P* = 0.894, Figure 2A), TNM stage (1+2 VS 3+4: 4.48±2.92VS3.90±2.99 , *P* = 0.196, Figure 2C), tumor size (<5 cm VS ≥5 cm: 3.96±2.90VS 4.42±3.03, *P* = 0.294, Figure 2D), tumor subtypes (intrahepatic VS extrahepatic: 4.42±3.03VS 3.82±2.84, *P* = 0.182, Figure 2E), vascular invasion (with VS without: 3.96±3.05 VS 4.32±2.91, *P* =0.431, Figure 2F), HBV infection (negative VS positive: 4.15±3.02 VS 4.27±2.81, *P* = 0.808, Figure 2G) and liver cirrhosis (with VS without: 4.41±2.62 VS 4.14±3.02, *P* = 0.663, Figure 2H).

**Table 1:** Beclin 1 expression status in relation to patient characteristics.

**Characteristics**	**No. patients (%)**	**Beclin 1 expression level**		***P* value**
		Low	High	
**Age (yrs)**				
<56	91 (50.6)	64	27	0.999
≥56	89 (49.4)	62	27	
**Gender**				
Male	115 (63.9)	79	36	0.735
Female	65 (36.1)	47	18	
**Subtypes**				
Intrahepatic	106 (58.9)	72	34	0.511
Extrahepatic	74 (41.1)	54	20	
**Largest tumor size**				
≤5 cm	95 (52.8)	67	28	0.872
>5 cm	85 (47.2)	59	26	
**Vascular invasion**				
Positive	69 (38.3)	48	21	0.999
Negative	111 (61.7)	78	33	
**HBV infection**				
Positive	44 (24.4)	28	16	0.344
Negative	136 (75.6)	98	38	
**Liver cirrhosis**				
Negative	153 (85.0)	109	44	0.374
Positive	27 (15.0)	17	10	
**Cholecystolithiasis**				
Postive	34 (18.9)	24	10	0.999
Negative	146 (81.1)	102	44	
**Tumor surgery**				
R0	163 (90.6)	116	47	0.282
R1/R2	17 (9.4)	10	7	
**Tumor stage**				
T1+2	65 (36.1)	47	18	0.735
T3+4	115 (63.9)	79	36	
**Node stage**				
N0	114 (63.3)	75	39	0.129
N1	66 (36.7)	51	15	
**TNM stage**				
1+2	86 (47.8)	57	29	0.331
3+4	94 (52.2)	69	25	

HBV, Hepatitis B virus; TNM stage, Tumor-Node-Metastasis stage.

### Beclin 1 Expression and Survival Analysis

**Figure 3:**
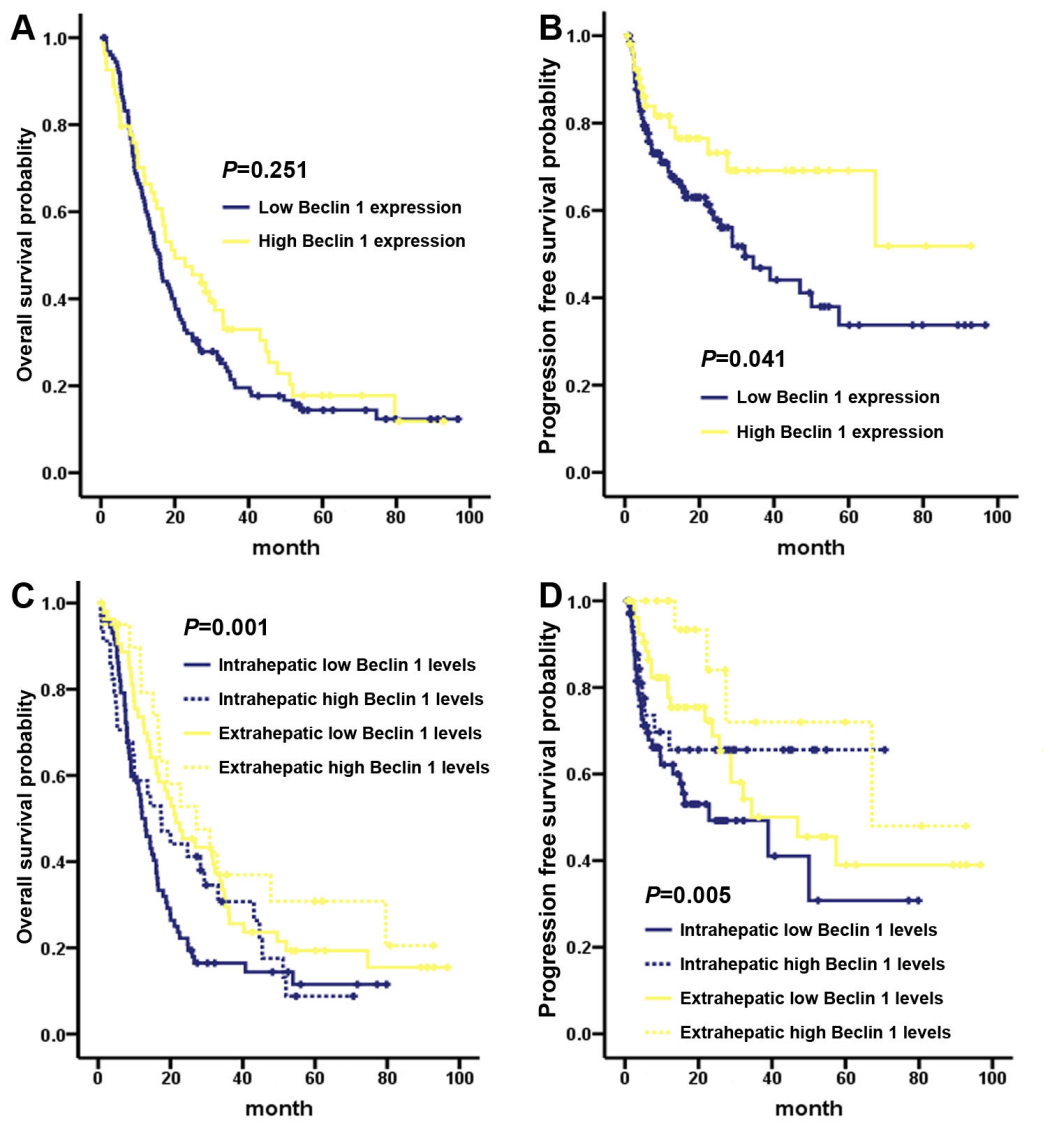
Overall survival and progression-free survival by Kaplan-Meier analysis, comparing the subgroup of patients with high or low Beclin 1 expression in cholangiocarcinoma. (A) Beclin 1 lowly and highly expressed patients had the similar overall survival. (B) Beclin 1 high expression predicted a favorable progression-free survival for cholangiocarcinoma patients. Stratified survival analysis showed that Beclin 1 expression level predicted a significant survival difference of overall survival (C) as well as progression-free survival (D) between ICC and ECC subgroups.

**Figure 4:**
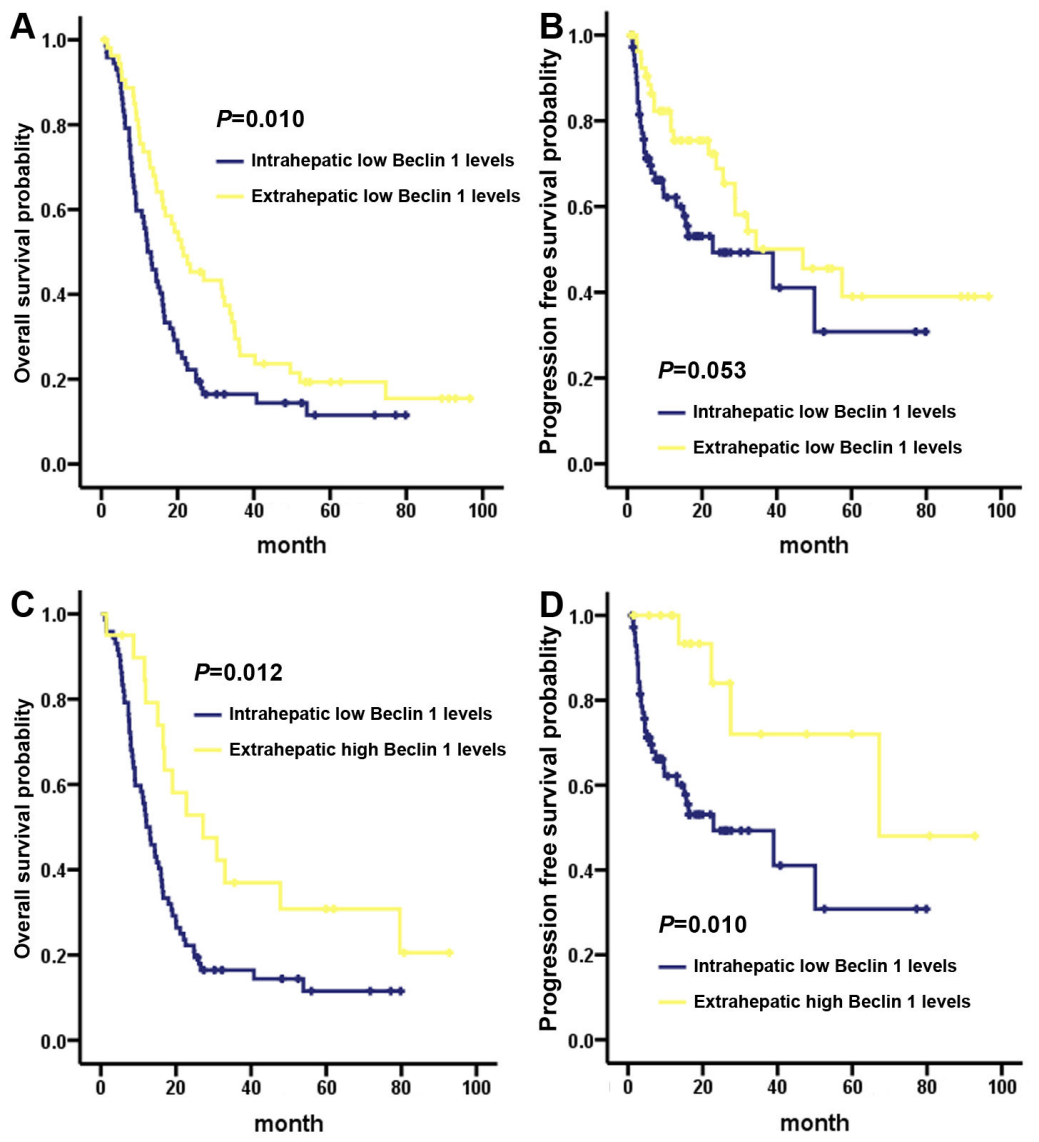
Stratified overall survival and progression-free survival by Kaplan-Meier analysis, comparing the subgroups of patients with high or low Beclin 1 expression in the subtypes of ICC and ECC. ICC subset patient with Beclin 1 low expression had an inferior overall survival (A) and progression-free survival (B) than that of Beclin 1 lowly expressed ECC subgroup patients. Compared to the ICC patients with Beclin 1 low expression, the ECC patients with Beclin 1 high expression had a superior overall survival (C) and progression-free survival (D).

We next tested the prognostic value of Beclin 1 in ICC and ECC. As shown in Figure 3, Beclin 1 high or low expression had the similar 3-year OS rate (high VS low: 21.4% VS 33.0%, *P* = 0.25) for the overall cholangiocarcinoma patients (Figure 3A), Beclin 1 high expression predicted a favorable 3-year PFS (high VS low: 69.1% VS 46.8%, *P* = 041, Figure 3B). Further stratified Kaplan-Meier analysis showed that Beclin 1 high expression predicted a better OS and PFS in ICC and ECC subgroups (OS, P = 0.001, Figure 3C; PFS, *P* = 0.005, Figure 3D). Significantly, the ICC subgroup with Beclin 1 low expression had a worsened 3-year OS rate than ECC subset (low Beclin 1 ICC VS low Beclin ECC: 16.5% VS 29.5%, *P* = 0.010, Figure 4A), especially than the Beclin 1 highly expressed ECC patients (low Beclin 1 ICC VS high Beclin ECC: 16.5% VS 36.9%, *P* = 0.012, Figure 4C). Similarly, the ICC subset with Beclin 1 low expression had an inferior 3-year PFS rate than ECC patient (low Beclin 1 ICC VS low Beclin ECC: 49.3% VS 50.1%, *P* = 0.053, Figure 4B), particularly than the Beclin 1 highly expressed ECC patients (low Beclin 1 ICC VS high Beclin ECC: 49.3% VS 72.0%, *P* = 0.010, Figure 4D).

### Univariate and multivariate analysis

Univariate analyses of clinicopathological variables were shown in Table 2. Multivariate analysis summarized in Table 3 displayed that Beclin 1 expression level (*P* = 0.049; hazard ratio (HR), 0.124) and liver cirrhosis status (*P* = 0.014; HR, 0.013) were the independent prognostic biomarkers to predict PFS. Moreover, histological grade (*P* = 0.006; HR, 7.713) and TNM stage (*P* = 0.049; HR, 5.120) were the independent indicators to predict OS. When stratified the cholangiocarcinoma patients into low Beclin 1 ICC and high Beclin 1 ECC subgroups, multivariate analysis showed that Beclin 1 was an independent prognostic biomarker both in OS (*P* = 0.043; HR, 0.752) and PFS (*P* = 0.004; HR, 0.541) (Table 4). Moreover, the tumor surgery status was also identified as a potential predictor of OS (*P* = 0.024; HR, 2.332) as well as PFS (*P* = 0.002; HR, 4.345) for the subtypes of ICC and ECC patients (Table 4).

**Table 2:** Univariate Cox proportional-hazards analysis in the overall patients.

**Variable**	**PFS**			**OS**	
	**Hazard ratio (95% CI)**	***P* value**		**Hazard ratio (95% CI)**	***P* value**
**Beclin 1** (high vs. low)	1.833 (1.016-3.308)	**0.044**		1.233 (0.861-1.766)	0.252
**Age** (≥56 y vs. <56 y)	0.695 (0.426-1.133)	0.144		1.166 (0.844-1.613)	0.352
**Gender** (Male vs. Female)	1.459 (0.864-2.464)	0.157		0.984 (0.703-1.379)	0.927
**Subtypes** (Intrahepatic vs. Extrahepatic)	0.596 (0.362-0.984)	**0.043**		0.614 (0.439-0.859)	**0.004**
**Largest tumor size** (≥5 cm vs. <5 cm)	1.934 (1.187-3.150)	**0.008**		1.903 (1.371-2.642)	**< 0.001**
**Vascular invasion** (Positive vs. Negative)	1.899 (1.168-3.086)	**0.010**		1.416 (1.014-1.977)	**0.041**
**HBV infection** (Positive vs. Negative)	1.620 (0.957-2.743)	0.072		1.139 (0.781-1.662)	0.498
**Liver cirrhosis** (Positive vs. Negative)	0.574 (0.312-1.056)	0.074		0.811 (0.518-1.267)	0.357
**Cholecystolithiasis** (Positive vs. Negative)	0.904 (0.474-1.727)	0.761		0.944 (0.614-1.452)	0.793
**Histological grade** (well/moderate vs. poor)	2.869 (1.508-5.458)	**0.001**		2.633 (1.714-4.043)	**< 0.001**
**Tumor surgery** (R0 vs. R1/R2)	3.465 (1.691-7.101)	**0.001**		3.095 (1.824-5.249)	**< 0.001**
**Serum CA199** (≥35 unit/ml vs. <35 unit/ml)	1.857 (0.867-3.979)	0.111		1.477 (0.931-2.342)	0.097
**Serum CA125** (≥35 unit/ml vs. <35 unit/ml)	1.844 (0.954-3.565)	0.069		1.679 (1.046-2.695)	**0.032**
**Serum CEA** (≥5 ug/L vs. <5 ug/L)	1.661 (0.911-3.029)	0.098		2.063 (1.394-3.053)	**< 0.001**
**Serum AFP** (≥8.1 ug/L vs. <8.1 ug/L)	1.096 (0.571-2.105)	0.783		1.019 (0.645-1.610)	0.936
**Serum total bilirubin** (≥23.9 umol/L vs. <23.9 umol/L)	0.762 (0.469-1.237)	0.272		0.801 (0.577-1.113)	0.187
**Tumor stage** (T1+2 vs. T3+4)	1.292 (0.781-2.139)	0.318		1.595 (1.131-2.249)	**0.008**
**Node stage** (N0 vs. N1)	1.330 (0.804-2.199)	0.266		1.416 (1.010-1.984)	**0.043**
**TNM stage** (1+2 vs. 3+4)	2.197 (1.332-3.623)	**0.002**		2.152 (1.541-3.006)	**< 0.001**

CA, cancer antigen; CEA, carcinoembryonic antigen; AFP, alpha-fetoprotein.

**Table 3:** Multivariate Cox proportional-hazards analysis in the overall patients.

**Variable**	**PFS**			**OS**	
	**Hazard ratio (95% CI)**	***P* value**		**Hazard ratio (95% CI)**	***P* value**
**Beclin 1** (High vs. Low)	0.124 (0.016-0.989)	**0.049**		0.555 (0.167-1.849)	0.338
**Age** (≥56 y vs. <56 y)	2.223 (0.388-12.733)	0.370		0.602 (0.190-1.911)	0.389
**Gender** (Male vs. Female)	1.032 (0.172-6.207)	0.972		0.717 (0.256-2.004)	0.525
**Subtypes** (Intrahepatic vs. Extrahepatic)	0.353 (0.031-4.002)	0.401		1.420 (0.252-8.019)	0.691
**Largest tumor size** (≥5 cm vs. <5 cm)	2.176 (0.181-26.195)	0.540		3.678 (0.641-21.122)	0.144
**Vascular invasion** (Negative vs. Positive)	0.139 (0.013-1.435)	0.098		0.767 (0.197-2.991)	0.702
**HBV infection** (Positive vs. Negative)	0.175 (0.011-2.869)	0.222		0.368 (0.049-2.781)	0.332
**Liver cirrhosis** (Negative vs. Positive)	0.013 (0.001-0.410)	**0.014**		1.119 (0.110-11.361)	0.924
**Cholecystolithiasis** (Positive vs. Negative)	0.451 (0.047-4.369)	0.492		0.384 (0.085-1.744)	0.215
**Histological grade** (Poor vs. Well/moderate)	3.443 (0.411-28.810)	0.254		7.713 (1.778-33.459)	**0.006**
**Tumor surgery** (R0 vs. R1/R2)	8.064 (0.345-188.626)	0.194		0.993 (0.128-7.721)	0.995
**Serum CA199** (≥35 unit/ml vs. <35 unit/ml)	0.591 (0.126-2.776)	0.505		2.004 (0.674-5.960)	0.211
**Serum CA125** (≥35 unit/ml vs. <35 unit/ml)	1.718 (0.355-8.318)	0.501		1.727 (0.640-4.659)	0.281
**Serum CEA** (≥5 ug/L vs. <5 ug/L)	2.727 (0.421-17.679)	0.293		1.011 (0.321-3.186)	0.985
**Serum AFP** (≥8.1 ug/L vs. <8.1 ug/L)	2.929 (0.411-20.892)	0.284		0.839 (0.225-3.128)	0.794
**Serum total bilirubin** (≥23.9 umol/L vs. <23.9 umol/L)	0.459 (0.102-2.062)	0.309		1.617 (0.636-4.114)	0.313
**Tumor stage** (T3+4 vs. T1+2)	9.733 (0.792-119.555)	0.075		1.124 (0.270-4.678)	0.872
**Node stage** (N0 vs. N1)	0.880 (0.098-7.906)	0.909		0.321 (0.079-1.307)	0.113
**TNM stage** (3+4 vs. 1+2)	1.658 (0.108-25.429)	0.717		5.120 (1.008-25.995)	**0.049**

**Table 4:** Multivariate Cox proportional-hazards analysis in ICC and ECC.

**Variable**	**PFS**			**OS**	
	**Hazard ratio (95% CI)**	***P* value**		**Hazard ratio (95% CI)**	***P* value**
**Beclin 1** (high vs. low)	0.541 (0.356-0.824)	**0.004**		0.752 (0.570-0.991)	**0.043**
**Age** (≥56 y vs. <56 y)	0.456 (0.199-1.043)	0.063		1.817 (1.057-3.122)	0.031
**Gender** (Male vs. Female)	1.766 (0.785-3.973)	0.169		0.833 (0.483-1.436)	0.510
**Subtypes** (Intrahepatic vs. Extrahepatic)	NA			NA	
**Largest tumor size** (≥5 cm vs. <5 cm)	NA			NA	
**Vascular invasion** (Negative vs. Positive)	1.967 (0.724-5.344)	0.184		0.894 (0.476-1.679)	0.728
**HBV infection** (Positive vs. Negative)	1.424 (0.613-3.308)	0.411		0.891 (0.458-1.732)	0.733
**Liver cirrhosis** (Negative vs. Positive)	1.737 (0.577-5.225)	0.326		0.880 (0.409-1.896)	0.745
**Cholecystolithiasis** (Positive vs. Negative)	0.568 (0.216-1.493)	0.251		0.911 (0.494-1.679)	0.765
**Histological grade** (well/moderate vs. poor)	NA			NA	
**Tumor surgery** (R0 vs. R1/R2)	4.345 (1.680-11.238)	**0.002**		2.332 (1.115-4.875)	**0.024**
**Serum CA199** (≥35 unit/ml vs. <35 unit/ml)	NA			NA	
**Serum CA125** (≥35 unit/ml vs. <35 unit/ml)	NA			NA	
**Serum CEA** (≥5 ug/L vs. <5 ug/L)	NA			NA	
**Serum AFP** (≥8.1 ug/L vs. <8.1 ug/L)	NA			NA	
**Serum total bilirubin** (≥23.9 umol/L vs. <23.9 umol/L)	1.530 (0.669-3.497)	0.313		0.919 (0.493-1.714)	0.791
**Tumor stage** (T3+4 vs. T1+2)	1.300 (0.498-3.394)	0.593		1.562 (0.847-2.882)	0.153
**Node stage** (N0 vs. N1)	1.390 (0.676-2.856)	0.371		1.800 (1.072-3.023)	0.026
**TNM stage** (1+2 vs. 3+4)	NA			NA	

## Discussion

The mammalian Beclin 1, the ortholog of yeast Atg6/Vps30, is an essential autophagic player that has been linked to diverse biological processes, including development, immunity, tumor suppression and lifespan extension [30,31]. Beclin 1 had been identified to be a novel prognostic biomarker in variety of solid tumors [22-24,26]. However, the phenotype of Beclin 1 and its prognostic value in intrahepatic cholangiocarcinoma (ICC) as well as extrahepatic cholangiocarcinoma (ECC) were unclear. Here, we detected Beclin 1 expression level in the subtypes of ICC and ECC, and in other pathophysiological contexts, such as HBV infection, liver cirrhosis and cholecystolithiasis. We found that, compared with normal adjacent tissues, Beclin 1 was lowly expressed in tumor zone both in ICC and ECC (Figure 1). In lymph node negative (N0) cholangiocarcinoma subgroup, Beclin 1 was relatively higher expressed than that of lymph node positive (N1) subset (Figure 1D and 2B). Moreover, Beclin 1 high expression was correlated with a favorable PFS for cholangiocarcinoma. Importantly, the stratified survival analysis demonstrated a significant OS as well as PFS difference in Beclin 1 high and low expression subgroups under diverse subtypes (ICC VS ECC, Figure 4). Thus, our study proved that Beclin 1 inactivation might facilitate an increasing in malignant phenotype, and a reducing in postoperative outcome for the subtypes of ICC and ECC.

Autophagy can be tumor suppressive by eliminating the oncogenic protein substrates, toxic proteins and damaged organelles [13]. Conversely, autophagy can also be tumor promotive through recycling of autophagic degraded cellular organelles to meet cellular metabolism necessary under nutrient starvation, hypoxia or other therapeutic stress [13,32]. Accordingly, as the central player of autophagy, Beclin 1 had a double-edged function in regulating tumorigenesis and other malignant phenotypes [33,34], and in predicting the prognosis for different tumor types [32,35]. We and others previously reported that, though head and neck squamous cell cancers (HNSCC) share the similar pathophysiological features, the prognostic significance of Beclin 1 was varied among the HNSCC subtypes [32,36]. For esophageal squamous cell carcinoma, an evident higher 3-year OS rate (near to 63.0%) was observed in Beclin 1 positive subgroup, particularly in the patients with HIF-1α low expression context [36]. However, our study found that overexpression of Beclin 1, positively correlated with HIF-1α level, predicted a poor OS (73.8% VS 84.5%), PFS (61.5% VS 79.5%) and distant metastasis-free survival (64.5% VS 84.7%) for nasopharyngeal squmous cell carcinoma [32]. These results indicated that, even share the similar biological behavior and anatomical characteristics, the prognostic phenotype of Beclin 1 might be distinguished among the tumor subtypes. Since the epidemiological and clinicopathological features are distinct between the subtypes of ICC and ECC [37], identifying more novel molecular biomarkers may allow better understanding the intrinsic nature of ICC and ECC. For ICC, previous study reported that Beclin 1 was strongly expressed in 24.1% (26/108) of overall patients, and Beclin 1 low expression was correlated with an inferior 3-year OS (22.4 VS 35.6%) and PFS, though the subsequent Cox regression analysis confirmed that Beclin 1 was not an independent indicator to PFS [26]. Consistently, we found that Beclin 1 was highly expressed in 32.1% (34/106) ICC and 27.0 (20/74) ECC (Table 1). Interestingly, our stratified survival analysis found that ICC with Beclin 1 low expression was correlated to a worsened OS and PFS than ECC subtype patients, especially than the Beclin 1 highly expressed ECC patients (Figure 4), suggesting that Beclin 1 might be a promising biomarker to select particular patients with high risk to death and disease progression in the subtypes of ICC and ECC. More importantly, this interesting finding might raise potential clinical application in determining the therapeutic regimen for cholangiocarcinoma: (1) for Beclin 1 lowly expressed cholangiocarcinoma patients, especially for ICC patients that had a poor OS and PFS, aggressive postoperative adjuvant chemotherapy might be necessary, and would be of survival benefit; (2). for Beclin 1 highly expressed cholangiocarcinoma patients, particularly for ECC subgroup that had favorable OS and PFS, postoperative adjuvant chemotherapy might mean overtreatment to these patients. 

Recent studies had proved that Beclin 1 is required for tumorigenesis, proliferation and motility [38]. The autophagic and oncogenic phenotype of Beclin 1 might be attributed to its interaction with class III phosphatidylinositol 3-kinase (PI3KC3) complexes and other important cellular regulators, including mammalian target of rapamycin (mTOR) [39], Bim [40], Bcl-xL [41], c-Jun N-terminal kinase (JNK) [42], Ambra 1 [43], Bif-1 and Beclin 1-associated autophagy-related key regulator (Barkor) [44,45]. Under the nutrient-deprived stress, mesenchymal stem cells (MSCs) survived by activating Beclin 1 mediated autophagy, and facilitated breast cancer MCF-7 cells to invasiveness through upregulating histone deacetylases 6 (HDAC6) activity and increasing motility [45]. Here, we found that the cholangiocarcinoma patients with lymph node metastasis (N1 stage) had a lower level of Beclin 1 than that of N0 stage patients (IHC score: 3.45±2.82 VS 4.60±2.97, Figure 2B). Consistent to 36.7% (66/180) patients staged to N1 in the present study, previous study reported that Beclin 1 was reduced expressed in primary ICC and the matched metastatic lymph nodes (29.6%, 32/108) [26]. Combined with the finding in ICC [26], our data further demonstrated that the correlation between Beclin 1 low expression and lymph node metastasis was indeed existed both in ICC and ECC. These findings indicated that Beclin 1 inactivation might be a frequent risk factor to lymph node invasiveness regardless of intraheptic or extrahepatic cholangiocarcinoma. Indeed, accumulated studies had confirmed the relationship between Beclin 1 and tumor metastasis in a series of tumors. In gastric carcinomas, decreased Beclin 1 was correlated with a poor differentiation, lymph nodal and distant metastasis [46]. Cheng et al. found that, compared with the normal cervical tissues and CIN tissues, Beclin 1 was lowly expressed in tumor, and closely correlated with lymph node metastasis for cervical carcinoma [47]. Similarly, increased Beclin-1 expression was closely correlated with the absence of local lymphatic invasion and low rate of distant metastasis for pancreatic ductal adenocarcinoma [48]. For colorectal and esophageal squamous carcinoma, the significant associations between Beclin 1 level and vascular invasion as well as lymph node metastasis were observed [36,49]. Those combined results confirmed that Beclin 1 was indeed involved in lymph node metastasis and other malignant phenotype for tumors. 

In conclusion, our study confirmed that Beclin 1 low expression, a negative prognostic indicator, was correlated with lymph node metastasis for cholangiocarcinoma. Combined Beclin 1 expression with tumor location would lead to a more accurate prognosis prediction for the subtypes of ICC and ECC.
